# Surgical Treatment of Carcinomas of the Oral Minor Salivary Glands—Oncological Outcome in Dependence of Tumor Entity and Therapeutic Strategies

**DOI:** 10.3390/cancers15153895

**Published:** 2023-07-31

**Authors:** Julius Moratin, Dominik Horn, Karl Semmelmayer, Oliver Ristow, Michael Engel, Jürgen Hoffmann, Moritz Bleymehl, Thomas Held, Sven Zittel, Christian Freudlsperger

**Affiliations:** 1 Department of Oral and Cranio-Maxillofacial Surgery, University of Heidelberg, Im Neuenheimer Feld 400, D-69120 Heidelberg, Germany; karl.semmelmayer@med.uni-heidelberg.de (K.S.); oliver.ristow@med.uni-heidelberg.de (O.R.); michael.engel@med.uni-heidelberg.de (M.E.); juergen.hoffmann@med.uni-heidelberg.de (J.H.); moritz.bleymehl@med.uni-heidelberg.de (M.B.); sven.zittel@med.uni-heidelberg.de (S.Z.); christian.freudlsperger@med.uni-heidelberg.de (C.F.); 2 Department of Oral and Cranio-Maxillofacial Surgery, Saarland University Medical Center, Kirrberger Straße, D-66424 Homburg, Germany; dominik.horn@uks.eu; 3 Department of Radiation Oncology, University of Heidelberg, Im Neuenheimer Feld 400, D-69120 Heidelberg, Germany; thomas.held@med.uni-heidelberg.de; 4 National Center for Tumor Diseases, Im Neuenheimer Feld 460, D-69120 Heidelberg, Germany; 5 Heidelberg Ion Beam Therapy Center, Im Neuenheimer Feld 460, D-69120 Heidelberg, Germany

**Keywords:** salivary gland tumor, salivary gland cancer, cervical metastases, neck dissection

## Abstract

**Simple Summary:**

Carcinomas of the minor salivary glands of the oral cavity are a rare and heterogeneous group of malignant tumors. The small number of patients limits the available data on treatment and outcome. In this study, we compared clinical and pathological features and oncological outcomes in a cohort of patients suffering from different kinds of minor salivary gland cancer who received primary surgical therapy. Overall, we found different rates of cervical metastases and disease recurrence in dependence on the tumor entity. Therefore, we conclude that the surgical therapy of patients suffering from minor oral salivary gland cancer is feasible and brings good oncological results although the different tumor entities require different levels of therapeutic aggressiveness and adjuvant treatment.

**Abstract:**

The aim of this study was to analyze the clinical outcomes of three types of minor salivary gland carcinomas (adenoid-cystic carcinomas (ACC), adeno carcinomas not otherwise specified (AC-NOS), and mucoepidermoid carcinomas (MEC)) after primary surgical therapy. A retrospective cohort study was designed and patients with cancer of the minor oral salivary glands treated in our department in the years 2011 to 2022 were included. Clinicopathological data were evaluated to compare overall survival and progression-free survival between the entities. Eighty-one patients were included. The rates of cervical metastases were 38.9% for ACC, 25% for MEC, and 9.1% for AC-NOS. ACC exhibited significantly higher rates of local and systemic disease recurrence (*p* = 0.02), and the presence of neck node metastases was confirmed as an independent prognostic factor for progression-free survival (*p* = 0.014). Treatment success in terms of oncological outcome varied significantly between the different entities and implies different treatment regimens for each tumor entity.

## 1. Introduction

Malignant tumors of the salivary glands are rare and only account for about 1–5% of head and neck cancers [1,2,3]. While those tumors are often included in the group of head and neck cancer, they are characterized by great clinical and molecular heterogeneity [4]. Thus, they differ significantly from squamous cell carcinomas, which are the predominant tumor type in this localization regarding pathogenesis and clinical course [5]. Many different entities form part of this group, including adenoid cystic carcinoma (ACC), mucoepidermoid carcinoma (MEC), and adenocarcinoma not otherwise specified (AC-NOS) as the most common malignant salivary gland tumors [6,7,8]. While most tumors are located within the three major salivary glands, only a small fraction of about 10–20% develops within the minor salivary glands that can be found mainly in the oral cavity [9,10,11]. Although there is an overweight of benign salivary gland neoplasms in total, several studies have shown that malignant tumors are more common in the minor salivary glands than in the major salivary glands [12]. They often present as painless swellings and, given the frequent localization at the hard palate, are diagnosed in advanced stages when osseous structures like the maxilla are already infiltrated [13].

Due to the heterogeneity and the rarity of those tumors, there is a lack of data on large cohorts, and treatment subsequently often is based on institutional standards, practitioner’s choice, or according to guidelines of other malignancies of the same localization, e.g., squamous cell carcinomas of the oral cavity. In most centers, primary therapy, thus, includes surgical resection with adjuvant radiotherapy if certain histopathological risk factors are present or primary radiotherapy in cases of irresectable tumors [14].

The treatment of the clinically negative neck (cN0) remains controversial, as data on cervical metastases are rare and often vary significantly between different publications and tumor entities [13,14]. The rate of cervical metastases, however, is crucial for treatment planning and patients’ outcome, as they have been identified as highly relevant prognostic factors in various head and neck tumors [13,15].

Thorough validation of the success of different treatment regimens and the consecutive clinical courses and outcomes are highly necessary to determine the optimal therapies for patients suffering from salivary gland carcinomas to achieve optimal oncological results and to avoid over- or under-treatment at the same time. The purpose of this study, thus, was to compare treatment modalities, pathological and clinical characteristics, and oncological outcomes in a cohort of patients suffering from adenoid cystic carcinoma, adenocarcinoma, and mucoepidermoid carcinoma of the oral minor salivary glands to exemplify relevant differences that may be relevant for treatment planning.

## 2. Materials and Methods

### 2.1. Patient Collective and Obtained Data

All patients with histologically confirmed diagnoses of carcinomas of the minor salivary glands of the oral cavity and primary surgical treatment in the department of oral and craniomaxillofacial surgery of the Heidelberg University Hospital between the years 2011 and 2022 were included in this analysis. In a second step, patients suffering from extremely rare tumors, or tumors that could not be sufficiently specified by the examining pathologist, were excluded to provide three homogenous groups of adenoid cystic carcinomas (ACC), adeno carcinomas not otherwise specified (AC-NOS) and mucoepidermoid carcinomas (MEC).

This study was approved by the local ethics committee (S-334/2018) and written informed consent was obtained from all patients. The digital patient recordings were reviewed using SAP patient management software (SAP, Walldorf, Germany), and the obtained data included clinical and pathological tumor stage, surgical procedures, perioperative management, duration of treatment and hospitalization, adjuvant therapy, and clinical follow up, including survival data. All available pathological reports were reviewed to obtain relevant histological data. Those included tumor grading, presence of histological risk factors (perineural, angio-lymphatic invasion), rates of cervical metastases, and resection status.

Overall survival was defined as interval from primary treatment to last clinical follow-up (censored data) or confirmed date of death. Progression-free survival was defined as interval from primary treatment to last clinical follow-up without progression (censored data) or confirmed date of local, regional, or distant disease progression.

### 2.2. Statistics

Analysis was performed with SPSS version 25.0 (SPSS Inc., Chicago, IL, USA). Descriptive statistics were used to illustrate demographic and clinical features of the investigated cohort. Mean values were compared using analysis of variance (ANOVA), and distributions were analyzed using chi-squared testing. The Kaplan–Meier method and log-rank testing were used to create survival plots and to determine differences between the groups. Multivariate Cox proportional hazard models were used to identify factors independently associated with overall and progression-free survival, including factors with significant prognostic impact in univariate analysis. A *p*-value of less than 0.05 was considered to be significant.

## 3. Results

### 3.1. Patient Cohort

An overall number of 81 patients with a mean age of 54.9 ± 15.8 were included in this analysis. A total of 41 patients (50.6%) were female, and 40 (49.4%) were male. ACC was the most frequent entity with 43 (53.1%) patients, followed by MEC with 24 patients (29.6%) and AC-NOS with 14 patients (17.3%). Table 1 provides demographic and clinical features of the investigated cohort.

The mean patient ages differed significantly with 54.7 ± 14.8 years in the ACC group, 64.4 ± 12.8 years in the AC-NOS group, and 49.8 ± 17.3 years in the MEC group. The gender distribution was equal for all three entities (Table 2).

### 3.2. Tumor Stage and Cervical Metastases

**Figure 1:**
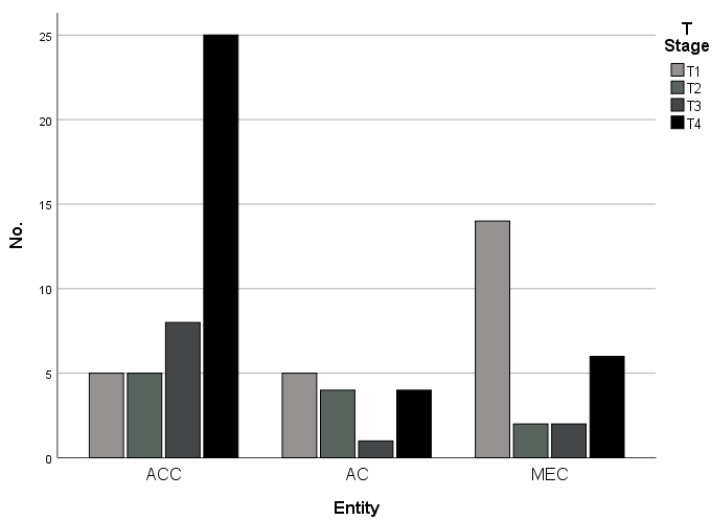
Distribution of T Stages (T1-T4) in dependence of tumor entity (adenoid cystic carcinomas (ACC), adenocarcinoma not otherwise specified (AC-NOS), and mucoepidermoid carcinomas (MEC)).

All tumors of the investigated cohort were staged according to the TNM Classification system of the oral cavity. The pathological tumor stages differed significantly between the different entities and demonstrated an overweight of T4 tumors in patients suffering from adenoid cystic carcinomas, indicating high rates of osseous tumor infiltration (Table 2 and Figure 1).

**Figure 2:**
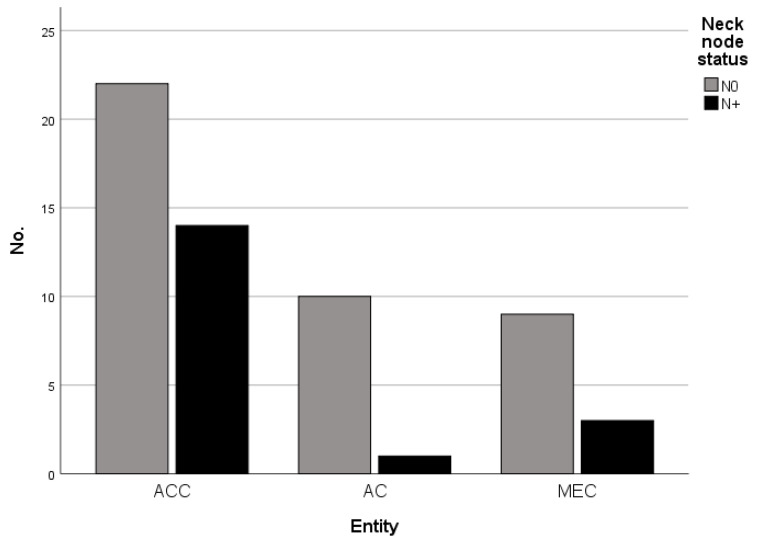
Occurrence of neck node metastases (N0 vs. N+) in dependence of tumor entity (adenoid cystic carcinomas (ACC), adenocarcinoma not otherwise specified (AC-NOS), and mucoepidermoid carcinomas (MEC)).

The rate of pathologically confirmed cervical metastases in the whole cohort was 22.2% (18 patients) and differed between the tumor entities (Table 2 and Figure 2). Although the differences were not statistically significant, ACC exhibited the highest rates of cervical metastases with 38.9% compared to 25% in MEC and 9.1% in AC-NOS (*p* = 0.154). There was a significant accumulation of positive perineural tumor invasion (PN1) in ACC in our cohort (*p* > 0.001), while the rates of lymphatic (L1, *p* = 0.073) and vascular (V1, *p* = 0.751) invasion did not differ significantly between the investigated entities.

### 3.3. Therapeutic Procedures

**Figure 3:**
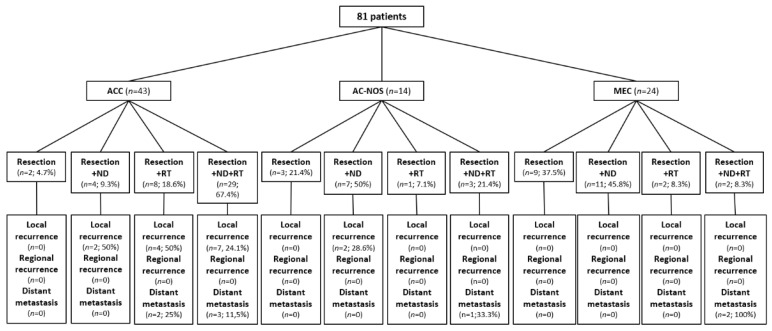
Flow chart depicting therapeutic procedures and rates of disease recurrence of 81 patients with carcinomas of the minor salivary glands of the oral cavity (ND—Neck Dissection; RT—Radiotherapy; ACC—adenoid cystic carcinoma; AC-NOS—adenocarcinoma not otherwise specified; MEC—mucoepidermoid carcinoma).

The therapeutic procedures executed in the presented cohort included sole tumor resection in 14 patients (17.3%), tumor resection with elective neck dissection (ipsi-/bilateral) in 22 patients (27.2%), tumor resection with adjuvant radiotherapy in 11 patients (13.6%), and tumor resection with elective neck dissection (ipsi-/bilateral) and adjuvant radiotherapy in 34 patients (42%). Figure 3 provides a flow chart depicting the different therapeutic regimens with consecutive clinical outcomes in dependence on the different tumor entities.

**Figure 4:**
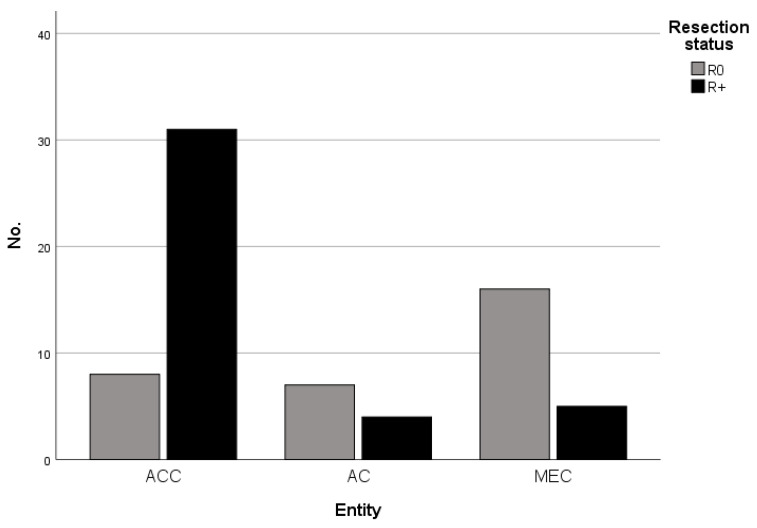
Resection status (R0 vs. R+) in dependence of tumor entity (adenoid cystic carcinomas (ACC), adenocarcinoma not otherwise specified (AC-NOS), mucoepidermoid carcinomas (MEC)).

Tumors limited to soft tissue received a resection according to the specific localization including clinical safety margins. In tumors with clinical and/or radiological suspicion or evidence of osseous infiltration (e.g., maxilla or mandible) and those tumors with close vicinity to osseous structures, the resection was extended to the neighboring bone. In all investigated malignancies, the achievement of clear resection margins (≥5 mm) was aspired. The comparison of the resection status revealed significant differences between the tumor entities with the highest rates of unclear or incomplete resection (R+) in ACC with 79.5% (31/39 patients) compared to 36.4% (4/11 patients) in AC-NOS and 21.7% (5/23 patients) in MEC (*p* < 0.001, Table 2 and Figure 4). In eight patients, reliable data on the resection status was missing and subsequently could not be included in the analysis. Correspondingly, the rate of postoperative adjuvant radiotherapy differed significantly between the entities with overweight in ACC (Table 2).

### 3.4. Disease Recurrence and Survival

The mean follow-up time in the investigated cohort was 51 ± 33 months, ranging from 4 to 123 months. Follow up included clinical examination and follow-up imaging via CT or MRI scans.

In total, 10 patients (12.3%) developed local tumor recurrence, 7 patients (8.6%) developed distant metastases and 5 patients (6.2%) developed both local and systemic disease recurrence during follow-up. In the 15 patients with local tumor recurrences, 1 patient received the best supportive care, 5 patients received tumor resection, 8 patients received definitive radiotherapy or re-radiotherapy, and 1 patient received resection with adjuvant radiotherapy. Distant metastases were treated with chemotherapy in dependence on general condition or resection of metastases in an individual approach in cases of singular metastasis and stable disease.

Table 3 provides an overview of the different types of disease recurrence dependence of the tumor entity.

**Figure 5:**
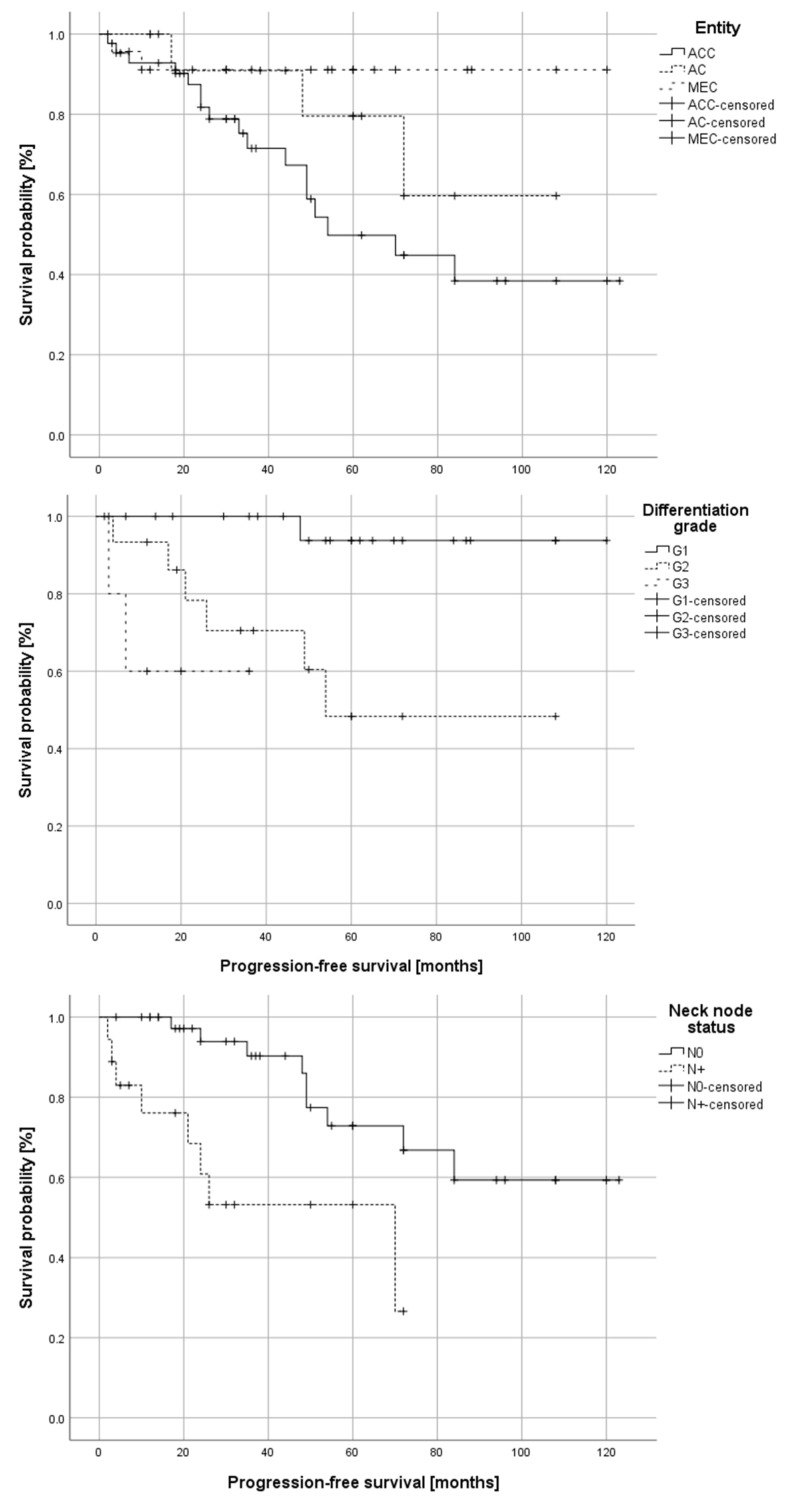
Kaplan–Meier plots depicting progression-free survival in 81 patients with carcinomas of the minor salivary glands of the oral cavity (ACC- adenoid cystic carcinoma; AC—NOS—adenocarcinoma not otherwise specified; MEC—mucoepidermoid carcinoma) in dependence of tumor entity (log-rank test: *p* = 0.032), tumor differentiation grade (G1–G3; log-rank test: *p* = 0.001) and primary neck node status (N0 vs. N+; log-rank test: *p* = 0.003).

Patients suffering from ACC exhibited significantly higher rates of disease recurrence compared to patients with AC-NOS or MEC (*p* = 0.02). Correspondingly, patients suffering from ACC exhibited significantly worse progression-free survival (log-rank test: *p* = 0.032) compared to the other groups. Figure 5 illustrates the differences in progression-free survival in dependence on tumor entity, differentiation grade, and neck node status (N0/N+). The only factor with a significant impact on overall survival in univariate survival analysis was the neck node status (log-rank test: *p* = 0.003).

Multivariate survival analysis was performed for overall and progression-free survival, including all variables with significant prognostic impact in univariate analysis. While there were no features with an independent prognostic impact on overall survival, the neck node status was shown to be an independent prognostic factor for progression-free survival (Table 4).

## 4. Discussion

This present study aimed to compare the local control rates and overall survival in patients suffering from different malignant tumors of the oral minor salivary glands undergoing primary surgery-based therapy. Small cohorts, often collected over long periods of time, and heterogeneous tumor entities have impeded the establishment of reliable treatment recommendations for salivary gland tumors in general. Moreover, the clinical behavior of tumors of the same entities seems to differ significantly depending on histopathological and biological features, such as differentiation grade, tumor localization (i.e., major vs. minor salivary glands), and different signaling pathways [16,17,18,19,20,21,22]. While genetic alterations may be of future use as therapeutic targets, up to date, the primary therapy of malignant salivary gland tumors is dominated by surgery and/or radiotherapy [14].

While surgical resection of the primary tumor, potentially complemented by adjuvant radiotherapy has been described as a therapeutic standard algorithm, a recommendation regarding the treatment of the clinically negative neck remains vague and contradictory and a differentiation of treatment recommendations adapted to the different tumor entities is missing so far [14,23,24].

We present clinical and pathological data of a large cohort of patients suffering from three different entities of malignant salivary gland tumors (i.e., adenoid cystic carcinomas—ACC; adenocarcinomas not otherwise specified—AC-NOS; mucoepidermoid carcinomas—MEC). As there exist different major and minor salivary glands and there have been numerous reports on the different clinical and pathological behavior of tumors in dependence on their localization, in this analysis, we focused solely on tumors of the minor oral salivary glands. This selection may also influence the accumulation of tumors with affection of the jaw bones in our analysis.

There is no doubt that most salivary gland malignancies, including high-grade tumors, advanced tumors (T3/T4), and such with clinical signs of neck node metastasis, require aggressive therapies, including radical resection with neck dissection, adequate defect restoration, and adjuvant radiotherapy, as stated by several authors and as presented in our analysis [14,18,24,25,26,27,28,29].

The significantly differing rates of primary neck node metastasis and disease recurrence, however, suggest a differentiated approach concerning the different tumor entities. While the higher rates of cervical metastases in the ACC group compared to the AC-NOS and MEC groups may partly be explained by higher tumor stages in those tumors. Furthermore, there was a significant accumulation of intermediate and high-grade tumors (G2 and G3) in the ACC group (*p* < 0.001, see Table 2), indicating a more aggressive tumor biology that also could explain the differing rates of cervical metastases. The differentiation grade is known to be a crucial parameter in salivary gland cancer and has been reported to be a significant prognosticator by various authors [18,30,31]. This is reflected in our data, as we found significantly differing survival rates depending on the differentiation grade in our analysis. Furthermore, there was a correlation of grading with the development of cervical metastases in patients suffering from MEC in our cohort, which is in line with the data reported in the available literature [32].

On the one hand, a rate of primary neck node metastasis of 38.9% for adenoid-cystic carcinomas, including an occult metastasis rate of 20.8%, in our investigation seems to justify a statement in favor of elective neck dissection of cN0 ACC patients, similar to the treatment recommendations in patients suffering from head and neck squamous cell carcinomas [33]. This is supported by the high independent prognostic significance of the neck node status for progression-free survival presented in our analysis. Furthermore, the neck dissection represents both a therapeutic as well as a diagnostic procedure, as a thorough pathological examination, including the cervical lymph nodes, helps to guide adjuvant radiotherapy in order to optimize oncological outcomes. For instance, the therapeutic regimen in our department of radiation oncology does not include standard radiotherapy of the neck in ACC patients with cN0 status. Here, elective neck dissection could help detect occult metastases (20.8% in our cohort) and, thus, reveal a therapeutic target for adjuvant treatment that would not have been addressed otherwise. With 38.9%, the rate of cervical metastases in patients suffering from ACC in our cohort is higher than those reported by several other authors (rates between 9 and 30%) [5,16,25,34]. This may partly be explained by a tendency towards advanced tumors being treated in our department as a nationwide center. Another explanation might be the fact that most ACC received elective neck dissection in cases of cN0 status. Considering the rate of occult metastasis in our cohort (20.8%), this fact also may have contributed to a high detection rate of cervical metastases. This assumption is supported by the article of Amid et al. who reported similar rates of cervical lymph node metastases in a large multicentric cohort of ACC patients undergoing neck dissection [35].

On the other hand, there were no cases of neck recurrences (i.e., metachronous cervical metastases) in our cohort. These findings are in line with several other publications on the course of disease in patients suffering from ACC [34,36]. While this may partly be due to the fact that most patients received elective or therapeutic neck dissection and adjuvant radiotherapy during primary therapy, this fact is interesting considering the often-reported rates of regional recurrences in head and neck squamous cell carcinomas [15,37]. In contrast to squamous cell carcinomas, ACC patients hardly seem to develop late neck recurrences, which probably decreases the relevance of neck dissection in ACC. Obviously, there are relevant differences in disease progression and/or recurrence between squamous cell carcinomas and ACC that impede the implementation of categorical analog therapeutic schemes regarding surgical treatment, e.g., of the clinically negative neck. While there are several publications on the high rates of cervical metastases in ACC patients, others reported on the missing survival benefit of patients with clinically negative neck node status undergoing elective neck dissection, although data derived from prospective clinical trials are missing so far [34,35,38,39].

The low rates of cervical metastases and especially the superior progression-free survival of patients suffering from AC-NOS and MEC suggest a less radical approach regarding the clinically negative neck in those entities to avoid over-treatment. Here, a focus on therapeutic neck dissection in cases of suspected neck node metastasis, as stated by other authors, seems reasonable and is in line with our data [14,16].

Interestingly, we did not see a significant difference in overall survival between the three tumor entities. This may possibly be explained by the mean follow-up period of 51 months. Several authors reported on the long clinical course of salivary gland tumors, and especially ACCs show a tendency towards late local and systemic disease recurrence after long periods of stability with late distant metastases being a main reason for tumor-dependent death in ACC patients [40,41]. A longer follow up, therefore, may be necessary to discover a possible difference in overall survival in the presented cohort. Progression-free survival, however, did show significant differences depending on the different tumor entities. This fact suggests different levels of aggressiveness regarding primary therapy and adapted follow up rather than a general therapeutic concept for all salivary gland malignancies.

The rates of incomplete tumor resections in our analysis were 76.6% for the ACC group, 35.7% for the AC-NOS group, and 12.5% for the MEC group (*p* < 0.001). These rates are especially interesting considering the fact that although we generally aspire to the achievement of clear margins in all malignant lesions, the aggressive nature of ACCs in our department leads to an even higher tendency towards radical resection (including neighboring bone, etc.) compared to the other investigated tumor entities. Other authors also reported high rates of incomplete tumor resections, especially in ACCs, although most reported rates of incomplete resection range between 30 and 65% [16,42,43]. The elevated rates of incomplete resections in our cohort may possibly be explained by the higher percentage of advanced tumors (T4 stage) in our cohort (*p* = 0.002) compared to the mentioned studies. Nevertheless, clear surgical margins have been identified as relevant prognostic factors in a variety of studies, and their achievements should be the main goal of surgical tumor therapy [16]. However, in our analysis, resection status could not be confirmed as an independent prognostic factor.

The formerly mentioned rates of advanced tumors and incomplete tumor resections in our ACC group may explain the significant difference in the administration of adjuvant radiotherapy between the tumor entities. The combination of surgery and adjuvant radiotherapy has been shown to provide high rates of local tumor control and, as a consequence, may preserve the quality of life in affected patients even if distant metastases occur during long-term follow up [13,16]. Considering the often-reported late disease recurrences of patients suffering from ACCs, long-term local control may be the most relevant quality criterion for primary therapy [41].

Isolated local disease recurrence was seen in 10 patients, and local recurrence in combination with simultaneous systemic spread was seen in another 5 patients. Surgical resection in terms of salvage surgery was performed in six patients. The concepts of salvage surgery with adequate reconstruction in cases of resectable local tumor recurrences and palliative tumor resection in cases of local recurrences with synchronous systemic spread have been shown to be feasible and successful options to improve a patient’s quality of life by achieving local tumor control and restoring or preserving esthetics and functions of the involved organ systems in a variety of studies, mainly focused on head and neck squamous cell carcinomas (HNSCC) [13,24,41,44]. Analog to other tumor entities, e.g., HNSCC, the treatment planning, and decision making regarding salvage surgery should be performed in specialized centers under thorough consideration of the affected patient’s individual wishes and capacities [45,46,47]. Again, the achievement of local tumor control while preserving or restoring the form and function of the orofacial system is crucial for affected patients in terms of quality of life.

## 5. Conclusions

In our analysis, we found relevant differences between the three investigated tumor entities regarding tumor stage at diagnosis, neck node metastasis, and clinical outcome in terms of progression-free survival. While the suspected higher aggressiveness of ACC compared to other salivary gland carcinomas seems to justify a more radical approach regarding surgery and/or radiotherapy, data on the use of elective neck dissection in cN0 patients stays controversial. Based on our clinical experience and the presented data, we, therefore, advocate a differentiated approach with more radical therapies in adenoid cystic carcinoma, while the high rates of progression-free survival in patients suffering from mucoepidermoid carcinoma suggest a more conservative approach in patients without clinical signs of metastasis.

## Figures and Tables

**Table 1:** Descriptive demographic and clinical data of the investigated cohort.

Parameter	Number of Cases (%)
Gender	
Female	41 (50.6)
Male	40 (49.4)
Age	
<20 years	2 (2.5)
21–40 years	13 (16)
41–60 years	31 (42)
61–80 years	29 (35.8)
>81 years	3 (3.7)
Tumor Localizations	
Hard palate	48 (59.3)
Soft palate	6 (7.4)
Mandible	11 (13.6)
Planum buccale	11 (13.6)
Tongue	2 (2.5)
Lip	3 (3.6)
Tumor Entities	
Adenoid cystic carcinoma (ACC)	43 (53.1%)
Adenocarcinoma not otherwise specified (AC-NOS)	14 (17.3%)
Mucoepidermoid Carcinoma (MEC)	24 (29.6%)

**Table 2:** Results of the correlation analysis of relevant clinical and pathological features of the cohort with the three included tumor entities (adenoid cystic carcinomas (ACC), adenocarcinomas not otherwise specified (AC-NOS), mucoepidermoid carcinomas (MEC); *p*-values according to ANOVA or chi-squared testing, respectively).

	ACC	AC-NOS	MEC	*p*-Value
Mean patient age (years)	54.7 ± 14.8	64.4 ± 12.8	49.8 ± 17.3	0.02
Gender				
Male	21 (52.5%)	8 (20%)	11 (27.5%)	0.79
Female	22 (53.7%)	6 (14.6%)	13 (31.7%)	
Pathological tumor stage (T)				
T1	5 (11.6%)	5 (35.7%)	14 (58.4%)	0.002
T2	5 (11.6%)	4 (28.6%)	2 (8.3%)	
T3	8 (18.6%)	1 (7.1%)	2 (8.3%)	
T4	25 (58.2%)	4 (28.6%)	6 (25%)	
Grading (G)				
G1	0	8 (72.7%)	16 (84.2%)	<0.001
G2	12 (80%)	2 (18.2%)	2 (10.5%)	
G3	3 (20%)	1 (9.1%)	1 (5.3%)	
Neck node metastases (N)				
N0	22 (61.1%)	10 (90.9%)	9 (75%)	0.154
N+	14 (38.9%)	1 (9.1%)	3 (25%)	
Resection status (R)				
R0	10 (23.3%)	9 (64.3%)	21 (87.5%)	<0.001
R + (R1/2/X)	33 (76.7%)	5 (35.7%)	3 (12.5%)	
Perineural invasion (PN)				
PN0	13 (31.7%)	9 (64.3%)	19 (86.4%)	<0.001
PN1	28 (68.3%)	5 (35.7%)	3 (13.6%)	
Lymphatic invasion (L)				
L0	32 (76.2%)	13 (92.9%)	22 (95.7%)	0.073
L1	10 (23.8%)	1 (7.1%)	1 (4.3%)	
Vascular invasion (V)				
V0	38 (90.5%)	13 (92.9%)	22 (95.7%)	0.751
V1	4 (9.5%)	1 (7.1%)	1 (4.3%)	
Adjuvant radiotherapy (RT)				
No adjuvant RT	8 (18.6%)	10 (71.4%)	20 (83.3%)	<0.001
Adjuvant RT	35 (81.4%)	4 (28.6%)	4 (16.7%)	

**Table 3:** Rate and type of disease recurrence in dependence of tumor entity (adenoid cystic carcinomas (ACC), adenocarcinoma not otherwise specified (AC-NOS), mucoepidermoid carcinomas (MEC)).

	ACC	AC-NOS	MEC	*p*-Value
Disease recurrence	17 (77.3%)	3 (13.6%)	2 (9.1%)	0.02
Type of disease recurrence				
Local recurrence	8 (80%)	2 (20%)	0	
Distant metastases	4 (57.1%)	1 (14.3%)	2 (28.6%)	

**Table 4:** Multivariate Cox regression analysis of progression-free survival in a cohort of patients suffering from malignant tumors of the oral minor salivary glands in consideration of relevant clinical and pathological variables (T Stage—Tumor Stage; N0/N+—Neck node metastases; 95-CI—95% Confidence Interval).

	Hazard Ratio (95-CI)	*p*-Value
T Stage	3.1 (0.9–10.1)	0.067
N0/N+	14 (1.7–114.1)	0.014
Tumor entity	1.3 (0.5–3.5)	0.564
Differentiation grade	1.6 (0.3–8.0)	0.558
Patient age	1.1 (1.0–1.1)	0.098

## Data Availability

The data presented in this study are available upon request from the corresponding author.
